# Short-range Bluetooth monitoring method for tracking, monitoring and follow-up of low-mobility wild species, *Choloepus hoffmanni* sp.

**DOI:** 10.1242/bio.061754

**Published:** 2025-05-21

**Authors:** Ricardo Villalba-Briones, Grecia Robles, Eliana Belén Molineros

**Affiliations:** ^1^ Escuela Superior Politécnica del Litoral (ESPOL University), Facultad Ciencias de la Vida, Campus Gustavo Galindo, Km. 30.5 Vía Perimetral, EC090112 Guayaquil, Ecuador; ^2^ Sacha Project Foundation, Fundación Proyecto Sacha, Urdesa Norte Avenida 1 #110A, 090501 Guayaquil, Ecuador; ^3^ Atelids Project, Proyecto Atelidos, km 15 via Salitre, 07/17 Los Prados, Ecuador

**Keywords:** Bluetooth monitoring, Sloth, *Choloepus*, Tracking methodology, SBMM, Low cost

## Abstract

Wildlife monitoring provides essential information for research, management, and release of rehabilitated animals. A handmade backpack with a tracker connected to a smartphone through Bluetooth signal was used to track rescued and rehabilitated two-toed sloths, *Choloepus hoffmanni*. The design of the equipment consisted of a malleable structure with biodegradable sections to favor independent detachment and a tied Tile tracking device in the dorsal surface, which, combined with transects, constitute a short-range Bluetooth monitoring method (SBMM). An experiment was conducted to compare the detection success of two-toed sloth detections through direct observation and SBMM in a forest patch. Direct observation technique was unsuccessful at detecting the two-toed sloth (0/4); in contrast, all the trials with the SBMM registered the presence of the two-toed sloth (10/10), 70% managed to pinpoint the location between three trees, 50% located the tree where the animal was, and 40% were able to visually detect it. Additionally, four two-toed sloths (three rehabilitated, one translocated) were monitored *in situ* in forests, and the backpack persisted a total of 110 days. In 84.3% of the 70 SBMM *in situ* implementation events examined, observation of the animal was achieved. In all cases, the backpack was released without human intervention. This method facilitates low-cost detection of low-mobility animals, especially after rehabilitation.

## Teaser

**Summary:** A practical biodegradable backpack tracking system enables low-cost monitoring of rehabilitated two-toed sloths, offering an autonomous tool for wildlife research and conservation.

## INTRODUCTION

Monitoring tracking devices are instruments of high interest in fauna management as they provide important information about the location and movement of animals in their environment (Fischer et al., 2018; Paterson et al., 2021). Monitoring animals through tracking tools is helpful for gathering data at a population or individual level. Satellite-based Global Positioning System (GPS) or radiotelemetry techniques are the most common in this regard, and are utilized to research on ecological patterns, behavioral interactions, survival rates, and population dynamics, among others, and also to support the adaptation to the wild of rehabilitated and released animals (Bannister et al., 2020; Choperena-Palencia and Mancera-Rodríguez, 2018; Dennis and Shah, 2012; Joo et al., 2022; Peery and Pauli, 2012; Thomas et al., 2011). Unfortunately, this technology can be expensive, and lack of economic resources to meet the proposed objectives, such as wildlife monitoring, is a common problem in conservation initiatives and rehabilitation centers (Choperena-Palencia and Mancera-Rodríguez, 2018; Guy et al., 2013; Thomas et al., 2011). Certain devices are tied to animals (with collars or backpacks) and are intended to avoid any possible harm to the animal caused by incorrect design, selection of materials, or weight of the device, but these procedures are not always innocuous (Coughlin and van Heezik, 2014; Jewell, 2013; Peniche et al., 2011; Thomas et al., 2011). Additionally, the handling of the individual during attachment and detachment of the tracking devices can be difficult and cause risks to the animal's survival (Dennis and Shah, 2012; Jewell, 2013).

As of 2025, there are several tracking techniques for wildlife monitoring, and they are selected depending on the objective of the activity, type of information needed, and, inherently, the available economic resources (Rault and Doyle, 2016; Thomas et al., 2011). In addition, the habitat in which the animal is present directly influences the type of monitoring implemented, as well as the physical characteristics, behavior and ecology of the species (Joo et al., 2022). Some methodologies detect animals by registering thermal infrared radiation (body heat), acoustic signals (vocalizations), radar-reflective profiles related to body size, or visual signatures such as motion or image features. This information is registered by specialized devices, including thermal cameras, acoustic monitoring systems, radar sensors, and camera traps, respectively (Christiansen et al., 2014; Honda et al., 2025; Hüppop et al., 2019; Joo et al., 2022). Other approaches involve attaching tracking devices that emit signals to the animals; these signals are then received and interpreted by monitoring systems. Terrestrial animal movement is commonly tracked using such devices, which transmit information to receivers and are subsequently analyzed by researchers (Joo et al., 2022; Thomas et al., 2011). Collars and backpacks with satellite-based trackers send the exact location at a given time and are extensively used for various purposes, from calculating home ranges of the animals to describe habitat preferences among others (Joo et al., 2022). In most cases, GPS techniques are favored over other options for remote wildlife tracking due to their accuracy and extensive possibilities, but the cost of materials is high, as is the expertise needed for their proper use (Joo et al., 2022; Thomas et al., 2011). Accelerometers determine activity patterns in three axes and have been used to track arboreal mammals such as koalas, *Phascolarctos cinereus* (Joo et al., 2022; Ryan et al., 2013)*.* Alternatively, automated radio tracking is a traditional technique to locate but shows less accuracy than the GPS-based tracking. On the other hand, manually operated radio equipment can detect an animal's location, but the geolocation of the animal is limited to the field work duration (Joo et al., 2022; Thomas et al., 2011). The radio signals are received by radio receptors, and triangulation is used in practice to calculate the location of individuals of a wide array of species, such as arboreal three-toed sloths, *Bradypus variegatus* (Giné et al., 2015; Joo et al., 2022; Thomas et al., 2011). However, the described techniques require specialists for their use and an investment in materials that institutions may not be able to provide (Choperena-Palencia and Mancera-Rodríguez, 2018). Due to the cost of these tracking devices, additional radio or GPS-based services are offered to retrieve the devices but also increase the final cost of the activity. Otherwise, the collar or backpack can also be automatically released at a specified time. To include a biodegradable section facilitates the retrieval of the tracking device (Cliffe et al., 2023; Villalba-Briones et al., 2022). Other commercially available and economic GPS-based options (∼$175 US) are able to send effectively the location of the target animal remotely to a smartphone but are limited by the presence of proper phone network signal (Fischer et al., 2018), which is not always the case in the wilderness.

Nowadays, countless species are monitored, and their characteristics will shape the tracking methodology in terms of the technology used, design, size of the device, or the number of additional resources needed in the fieldwork (Thomas et al., 2011). Thus, knowledge of the species characteristics is prerogative to the tracking implementation. Two-toed sloths, *Choloepus hoffmanni*, are nocturnal animals that depend on their ability to move unnoticed to avoid predators (Suutari et al., 2010; Villalba-Briones et al., 2022). In this regard, sloths exhibit slow movements and have camouflage capacities (Suutari et al., 2010; Villalba-Briones et al., 2022). The two-toed sloths are placental mammals from the Xenarthra order that live in trees and feed on leaves, flowers, and fruits, but they also hunt opportunistically insects and small vertebrates (Duarte Riaño et al., 2016; Hayssen, 2011). They are mainly sleeping during the day, and they increase their activity from dusk to dawn, using relatively high trees and large emergent trees (Honda et al., 2025; Mendel, 1981; Sunquist and Montgomery, 1973; Villalba-Briones et al., 2022). Their movement is mostly restricted to branches of the same tree or nearest trees, but they also can move invertedly longer distances (Fernando Acevedo-Quintero et al., 2011; Sunquist and Montgomery, 1973; Villalba-Briones et al., 2022, 2023) and, occasionally, on the ground (Mosquera et al., 2019). Due to the vulnerability exhibited by two-toed sloths on the ground, they descend only once every 5-7 days to defecate and rarely walk on the ground to change tree or to ingest essential minerals and water through consumption of soil (Mendel, 1981; Stewart et al., 2022). The work of Sunquist and Mongomery (1973) on two-toed sloths' patterns of activity registered an average of 7.6 h of activity per day (*n*=25). The distances covered in their movement are usually limited: 54% of the animals exhibited at least 38 m movement per day, and 11% showed less than 7 m movement, indicating that no change in tree was performed on successive nights (Sunquist and Montgomery, 1973). In addition, two-toed sloths display a small home range [sample mean (X̄)=7.5 ha, range=0.6-101.1 ha] and low population density (Mendoza et al., 2015; Superina et al., 2010). On the contrary, in terms of distribution, they are widely spread through different countries of Central and South America, presenting differences that have contributed to establishing several subspecies (Hayssen, 2011; Plese et al., 2016). According to Hayssen (2011), the coast of Ecuador hosts the subspecies *C. hoffmanni capitalis*, a subspecies with a clearer whitish fur in the head area. This subspecies distribution is fitted in the area recognized as a hotspot for conservation (Chocó-Darien-East of Ecuador) due to its high degree of endemism and high degree of habitat loss (Myers et al., 2000; Williams et al., 2011). By reason of the limited research on this subspecies condition, in this work, we will refer to the subjects of this study as *C. hoffmanni*. Wild populations of Ecuadorian coastal areas suffer from multiple threats of anthropogenic origin such as deforestation; also, two-toed sloths are widely hunted species for illegal consumption of bushmeat in several Latin American countries (Martínez et al., 2020; Rivas et al., 2020; Superina et al., 2010; Villalba-Briones et al., 2023). Recently, two-toed sloth hunting has been reported in an Ecuadorian coastal region (Villalba-Briones et al., 2023). In addition, in Guayaquil, Ecuador, from the list of mammals subjected to fauna trafficking and bushmeat consumption (Ministerio del Ambiente de Ecuador, 2017), the two-toed sloths are the second most abundant species received by a veterinary center adapted for the treatment of wildlife, Mansion Mascota (20 individuals in 3 years; 12.1% of the total) (Villalba-Briones et al., 2021). Even if the conservation status of *C. hoffmanni* is defined as of least concern globally, populations are decreasing, and *C. hoffmanni capitalis* is categorized as vulnerable in Ecuador (Tirira, 2021). Unfortunately, there are still gaps in the knowledge on two-toed sloths, and further research is needed on relevant issues for conservation, such as life history, population dynamics and home range (Plese et al., 2016; Superina et al., 2010).

The survival rates of two-toed sloths during rehabilitation are low, and the monitoring of the released individuals after rehabilitation is scarce (Choperena-Palencia and Mancera-Rodríguez, 2018; Duarte Riaño et al., 2016; Villalba-Briones et al., 2022). The main objective of fauna rehabilitation is to return the animals to their habitat (Paterson et al., 2021). Within the factors influencing the survival rate of rehabilitated fauna release, the monitoring of the animal to evaluate its adaptation to the wild is recognized as beneficial (Cope et al., 2022; Pottie et al., 2021). Monitoring the released animals can give important information to detect weaknesses in rehabilitation and threats, and, most importantly, it can facilitate assistance to the animal during its establishment period (Pottie et al., 2021). The first weeks or months of release are critical in establishing animals in their surroundings (Pottie et al., 2021). Monitoring during establishment enhances a follow-up that facilitates assistance to the animal; thus, the animal can be aided in critical situations. It facilitates behavioral and general health assessments, direct nutritional support, and relocation or rescue of the individual in the worst-case scenario, which in the end, increases the success of the released animals (Pottie et al., 2021; Villalba-Briones et al., 2022). The follow-up of these animals without supporting tracking devices is of great difficulty and effort due to a combination of elusive behaviors, slow movements, and ability to camouflage in its natural environment (Choperena-Palencia and Mancera-Rodríguez, 2018; Mendel, 1981; Villalba-Briones et al., 2022). That is the reason why technically supported monitoring helps to check the animal's situation, but it is also useful for research on behavior and ecological needs, which again are essential to know for efficient rehabilitation and release (Mendoza et al., 2015; Pottie et al., 2021; Villalba-Briones et al., 2022).

In this work, we present the evaluation of a method designed to satisfy the need for monitoring at a low cost, as a response to the typical scarcity of conservation resources in terms of monitoring for this and other animals of low mobility, and, especially, for released animals after rehabilitation.

## RESULTS

### SBMM evaluation experiment results

None of the four-member teams performing non-assisted visual monitoring through linear tracks was able to detect or confirm the presence of the two-toed sloth in the area (0/4) (Table 1).

**Table 1.** General information related to the two-toed sloth (*Choloepus hoffmanni*) individuals monitored after release via short-range Bluetooth monitoring method (SBMM)

Individual	Origin	Time of rehabilitation	Weight at reception-release (kg)	Sex	Backpack conditioning period (days)
Bravo*	Orphaned	11 months	0.75-3.8	Male	54 and 14
Polita	Hunting	2 months	3.8-4.35	Female	5
Lina	Rescued	1 month	4.1-4.05	Female	15
Argal	Translocated animal	0	Not registered	Male	0

*Previously published in Villalba-Briones et al. (2022).

The use of the SBMM during monitoring activities allowed all the participants to confirm the presence of the two-toed sloth in the area (10/10): 4/10 visually detected the two-toed sloth, 1/10 successfully located the tree in which the two-toed sloth was without visual record, 2/10 located the two-toed sloth within a guess of two and three trees, respectively (Table 2), 1/10 failed in guessing the trees in which the two-toed sloth could be located, on 1/10 occasions the SBMM failed due to transitory loss of connectivity of the smartphone, and for 1/10 activity was suspended due to free abandonment of the practice (Table 2). The average visual detection time was 6.25 (±4.5) min.

**Table 2.** Results of the different groups that carried out monitoring of the two-toed sloths during the experiment

Method	Group type	*n*	Presence of sloth	Two to three tree locations	Tree location	Visual record
Visual transect	Group without experience	4	0	0	0	0
Short-range Bluetooth method (SBMM)	Group with experience	4*	4*	2	2	2
	Individual without experience	3^‡^	3^‡^	2	1	0
	Individual with experience	3	3	3	2	2
	SBMM total	10	10	7	5	4

*One stochastic error during experimentation due to technical non-detectability. ^‡^One suspension of practice due to free abandonment of the activity.

In the case of failure of the method due to lack of efficiency in portraying the intensity of the signal, as the smartphone detected the Tile's signal but not the intensity, the animal's presence in the area was confirmed but not its exact location. After restarting the cell phone and/or Bluetooth signal, the signal is usually restored. The range was systematically recorded only during the experimental trials, using GPS to measure the distances. According to the GPS data, the intensity of the signal was reliably detectable at variable distances, up to 35 linear meters from the two-toed sloth in plain terrain. This variation was primarily due to obstacles, such as leaves and branches, that the Bluetooth signal encountered, which directly impacted its range.

### *Inmsitu* monitoring of rehabilitated two-toed sloths results

All four individuals were detectable during monitoring under SBMM implementation. In all cases, the backpack was released autonomously without human intervention. The endurance of the backpack on the two-toed sloth varied dramatically, from 3 to 98 days (Table 3). The causes of release of the backpack were environmental issues (exposition to heavy rain in the case of Bravo and Lina) and independent release of the backpack by the two-toed sloth (Argal and Polita) (Table 3). Independent release of the backpack occurs when the sloths press against the backpack, detaching the Velcro junctions between the false leather arms and the cardboard area. In total, the two-toed sloths wore the backpack for 110 days, and 70 SBMM events in secondary dry tropical forests were implemented. In these events, 84.3% rate of success in observing the animal was accomplished. In all four monitored individuals (Bravo, Polita, Lina and Argal), the detection capability was high (66.6-100% success) (Table 3).

**Table 3.** Results from assessment of the SBMM applied in rescued (Bravo, Polita, Lina, Argal), rehabilitated (Bravo, Polita, Lina) and released two-toed sloths to forests

Individual	Days with the biodegradable backpack	Cause of cease of monitoring through SBMM	Success in visual detection by observation (days)	% of success in observation	Movement area (Ha)
Bravo*	6	Heavy rain	6/6	100	0.12
Polita	3	Independent release	3/3	100	0.16
Lina	98	Heavy rain	48/58	82.7	0.34
Argal	3	Independent release	2/3	66.6	0.08
Total	110	Heavy rain and independent release	59/70	84.3	0.69

*Continuous night monitoring and follow-up results published in Villalba-Briones et al. (2022).

In two cases (Bravo and Lina), the rain debilitated the cardboard area of the backpack so the tracking device could be released, inflicting no harm to the animal in the process. Bravo was released in a forest (Bosque Protector Prosperina-Guayaquil) located in a mountain range with sloping terrain exceeding 40% in February (Merchán-Sanmartín et al., 2023). The release of the animal was carried out during the rainy season, a period characterized by increased food availability for herbivores. Consequently, it is considered the least stressful time of the year for sloths (Dias et al., 2009). The rainy season in coastal Ecuador is characterized by extreme rainfall episodes that, in the area of study, generate floods and stationary rivers (Merchán-Sanmartín et al., 2023; Rollenbeck et al., 2022). In the case of Bravo, the mountain range climatology of the study site provoked lightning and heavy rains that generated sudden stational rivers around during monitoring. Consequently, after disintegration of the cardboard junction and release of the device, a visual follow-up of the animal was performed (for 9-11 h during the night). Thanks to this effort, the physical integrity of the animal was confirmed until the animal was no longer visually detected (Villalba-Briones et al., 2022). In the case of Argal only, the animal was sought 1 day after the loss of the backpack (Fig. 3D). In the rest of the cases, after the tracking device was released, no visual detection of the animal was accomplished, so the status of the animal after this moment could not be confirmed. In the case of Lina, the SBMM lasted more than 3 months and was able to verify supported and independent feeding during monitoring (Fig. 3B,C). In all cases, after the release of the backpack due to the disintegration of the cardboard junction, or individual release through induced force with the arms, the tracking device was recovered together with the backpack to be reutilized. The home range analysis revealed minimal displacement of the animals within the monitored period (ranging from 0.12 to 0.34 ha).

## DISCUSSION

Considering the low budget of rehabilitation centers and the subsequent scarce post-release follow-ups, the SBMM proves to be helpful for the monitoring of low-mobility species such as the two-toed sloth. The experiment, which involved testing direct observation through a linear transect method against the use of SBMM with 15 min of individual training (yielding 0/4 observations for the former and 4/10 observations for the latter, along with 1010 presence detections), demonstrated the effectiveness of the SBMM. At the same time, all two-toed sloths with *in situ* use of the SBMM were able to autonomously release the backpack, and no harm was detected in the animals. We can infer that the proper habituating to the backpack during rehabilitation provides longer endurance of monitoring, as the animals that were new to the device (Polita and Argal) were able to detach it independently after 2 and 3 days, respectively. The endurance of the backpack on the animal depends primarily on the resistance of the harness junctions and the materials used. Since animal well-being was prioritized, we selected a harness-type structure that is temporarily attached to the animal through a biodegradable cardboard component. Although this choice minimizes potential harm, it also affects durability. Additionally, environmental factors such as climatic uncertainty, the animal's behavioral response to rainfall, and prolonged exposure to humidity, can accelerate the degradation of the biodegradable junction. These combined environmental and behavioral factors make it difficult to precisely predict the durability of the SBMM. Consequently, the duration and success of monitoring events using this method can vary considerably. Nevertheless, with careful planning and strategic deployment, the SBMM can still effectively support the achievement of key research and conservation objectives. The cost of the fabrication of the backpack is below $40, and all backpacks were recovered in the days following the detach, which facilitates the reusability of the backpack upon replacing the cardboard junction and makes the SBMM available for repeated use. The home range of the animals monitored shows little movement compared with studies based on free animals; but, in three of four cases, were above the minimum values registered in the wild (X̄>0.1 ha) (Mendoza et al., 2015; Sunquist and Montgomery, 1973). The longer monitoring period enabled the most extensive use of the space; therefore, the lower values may be attributed to the shorter monitoring duration.

The use of the Tile app and Bluetooth technology has already been proposed in different fields, such as locating people with dementia (Shu and Woo, 2021). The simple use of the SBMM simplifies monitoring for inexperienced volunteers helping in locating animals. Thus, the SBMM enables caregivers to provide protection to and follow-up released animals, and, consequently, to support or rescue them if needed, which is highly recommended during the establishment of an individual in the habitat (Pottie et al., 2021). However, it is important to note the low range of detection of the Bluetooth technology of the Tile pro device (120 m in plain terrain with no obstacles), although there is no need for internet or phone signal, which is usually absent in the context of wild animals' release. Although the SBMM is far from matching the capacity of other methods such as radiotelemetry or satellite-based tracking and it does not substitute these methods, the technical skills needed and the costs are minimal in comparison. In the future, the improvement of Bluetooth or the inclusion of other low-budget tracking technology could strengthen and expand the detection features of backpacks, becoming part of the design. Additionally, new techniques such as thermo-imaging detection could be also utilized in combination with other methods to increase the detectability of cryptic animals (Pocknee et al., 2021).

To verify the correct function of the smartphone connection, a test with an extra tile is recommended, and map location (only if internet is available) and degree of intensity exposure should be checked before every attempt. The optional sound produced by the Tile during the search function was not activated during experimentation to avoid undesirable stress.

Knowledge on ethology and behavioral ecology of the species facilitates the creation of new species-specific techniques to maximize the well-being of the animals in captivity, as well as during rehabilitation, transport, and follow-up after release (Rault and Doyle, 2016). The efficiency of the SBMM is related to the physical and behavioral characteristics of the species. The sensitiveness of the animals must be considered in all activities designing adaptive responses to every step from the rehabilitation to the post-release monitoring. The handling of two-toed sloths should be slow and gentle, avoiding any perception of aggression, and should be adapted in response to the animal's behavior. Spending time progressively attaching the different segments could favor the acceptance of equipment. Slow movements and soft voice of the caregiver or a person with whom the animal is acquainted can minimize the stress on two-toed sloths. We recommend distracting the animal after fitting the backpack with preferred foods or new branches in the environment that will cause curiosity and gain the attention of the animal. Considering the vulnerability on the ground of these species, we also recommend adding a transversal wooden stick to the inside of the cage, enabling the animal to grip and perch during any transportation. After selecting an appropriate area, in the moment of the release, it would be gentle to open the cage close to a tree and patiently allow the animal to climb it under the watch of the facilitator.

In the SBMM, the collar or backpack design must have biodegradable sections to allow a passive detachment, so that the animal does not suffer stress unnecessarily at the moment of retrieving the device, avoiding captures and immobilizations (Dennis and Shah, 2012; Jewell, 2013). At the same time, to enable the SBMM to perform, it is necessary to combine the backpack tracking capacity with transects to get the location of the individual within the tile device detection range. It is recommended to start checking the Bluetooth signal in the app before arriving at the position of the last visual record of the detection of the animal to gain time. The tile detection range and signal intensity varied significantly depending on the obstacles present in the field, so it is also recommended to try in different directions to avoid all possible interferences. Any handling of wild animals will require a permit from the correspondent environmental institutions.

### Conclusions

The SBMM, which includes a biodegradable backpack with an integrated Bluetooth tracking device can be handcrafted and offers a practical solution for monitoring individual animals. This cost-effective approach proves to be a viable option for conservation efforts, research projects, and especially in rehabilitation programs. The SBMM facilitates supervision of the performance of the released animals, is light and comfortable on the animal's body, and provides a low-cost technique that can help caregivers and researchers to support fauna. This method can solve many of the problems in rehabilitation procedures related to the evaluation of post-release success. At the same time, the achievement of establishment to the natural environment of rehabilitated and released animals can be promoted by adding a follow-up activity to assist the animal in this first and high-risk period of independence after rehabilitation. To verify the acceptance of the backpack and to obtain longer periods of monitoring through SBMM, a period of habituating to the backpack is recommended during rehabilitation in captivity. Due to its effectiveness, availability, and simplicity, the SBMM is recommended to be incorporated into release protocols and utilized during specific periods for species that exhibit minimal movement and are challenging to detect in the wild, such as sloths (*Folivora* suborder), koalas (*Phascolarctos cinereus*), or loris (*Lorinae* subfamily) species.

## MATERIALS AND METHODS

### Description of the invention

The biodegradable and reusable innovation consists of a soft structure backpack that fits the animal's body with a cardboard junction. The backpack includes an integrated commercial Bluetooth tracker, so the whole device can be crafted at home with the appropriate materials. The ventral section has a cardboard structure where the four elongated arms adhere to complete the backpack structure. The adhesion is achieved by the Velcro segments sewed to the cardboard structure and to the end of the longitudinal stripes made of false leather, thus facilitating a junction and closing the backpack structure. Due to the cardboard segment, the backpack is detached under the influence of moisture and pressure, which varies depending on the season of the year and the action of the animal, respectively. Although the backpack's integrity is vulnerable to these effects, it can endure enough to allow the monitoring of the animal adequately. The Velcro sections facilitate the release of the backpack, under generation of a greater force by the animal independently. Velcro can be manually adjusted to animals of different sizes without anesthesia, due to the possibility of fixing it gently around its body, adding pressure on the Velcro of the different sections. At the same time, the commercial Bluetooth device is fixed in the rear by adhesive tape so that it is transported with the animal in the backpack. In our case, the tracking device used is the Tile^®^ Pro (2020) model, which operates via Bluetooth technology and connects to a smartphone. It emits a Bluetooth signal with a detection range of up to 120 m and features a replaceable battery designed to last up to 1 year (Tile Inc., San Mateo, CA, USA; www.thetileapp.com/en-us/). In this setup, the Tile device, fixed to the animal's backpack, sends its location via Bluetooth to the connected smartphone. The intensity of the signal displayed on the smartphone's screen is proportional to the proximity of the tracker, providing the most useful information to gradually approximate the animal's location. Consequently, the Tile app receives data about the animal's proximity, which helps in locating it within the available range of the Tile Pro device. To improve the precision of the tracking and enable the creation of detailed movement maps, a Garmin GPS is also employed. These additional GPS data provide accurate location points that are essential for calculating the animal's home range and mapping its movements (Villalba-Briones et al., 2022).

**Fig. 1.**
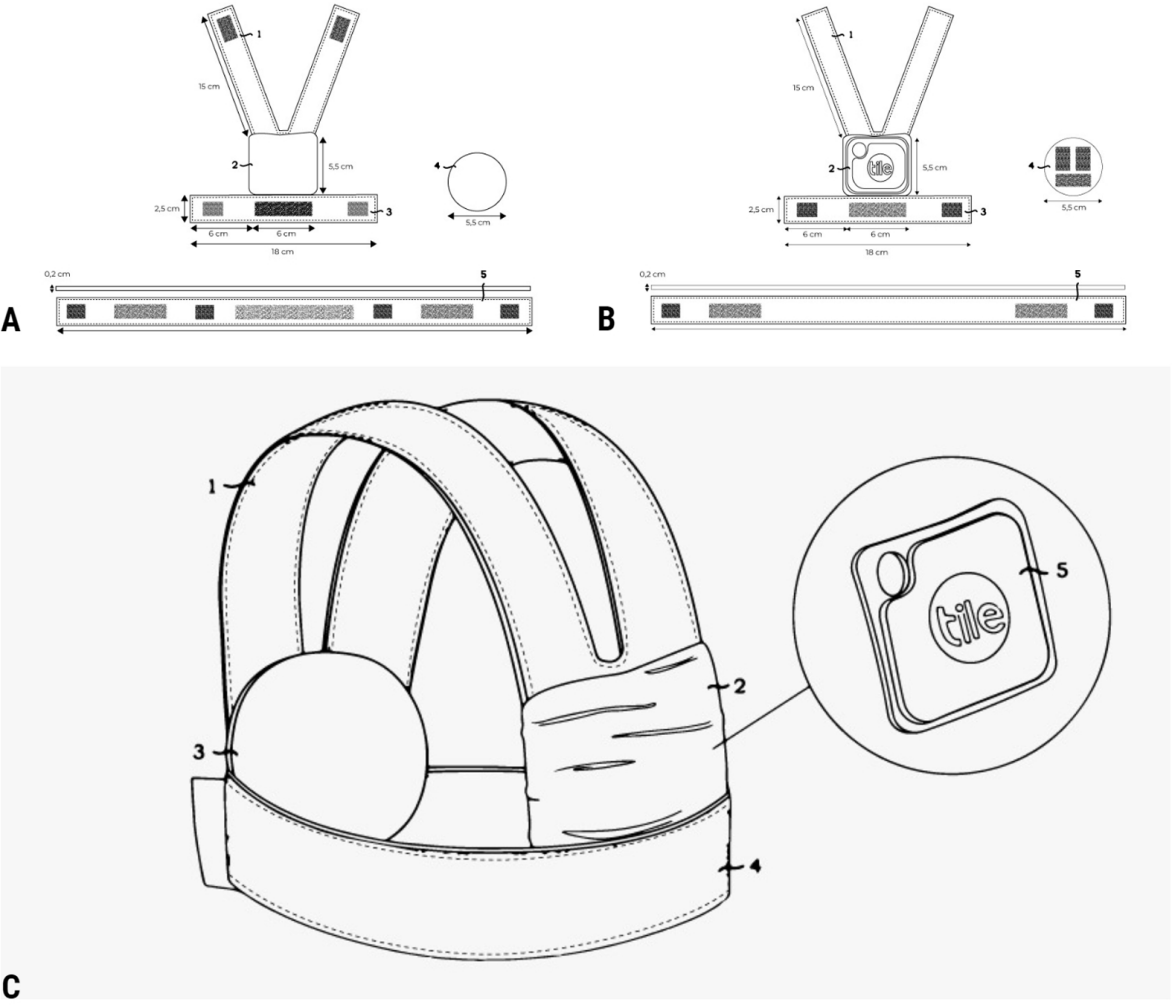
**Design of a backpack with a release via degradation of the junction used in the short-distance Bluetooth monitoring method (SBMM).** The size of the backpack should be decided to fit each individual. (A) Drawing of the dorsal view of the structure and the three components of the backpack. (B) Drawing of the ventral view structure and the three components of the backpack. (C) Drawing of the external view of the structure mounted and closed, with the ends of the backpack adhered to the central cardboard structure. Extracted from proposal for patent number SENADI-2023-58965.

The biodegradable backpack consists of two sections of false malleable leather, and a circular section of cardboard, with Velcro adhesive to join one to each other (Fig. 1A,B). This design is constituted by three different sections; the dorsal section contains the Tile pro device (Fig. 1C), the belt section consists in an elongated malleable leather stripe (marked as 5 in Fig. 1A,B), and a third ventral section consists of a circular junction core made of cardboard (marked as 4 in Fig. 1A,B) with Velcro adhesions for belt and dorsal sections (Fig. 1A,B). The Velcro is displayed as gray grids for the soft side and black Velcro elements for the rugged side, so that by pressing against each other, the adhesion of the different elements is achieved, and the backpack is closed (Fig. 1C).

The backpack fitted to the animal's body gets adhered by Velcro to hardened cardboard, which is covered with a thin layer of glue to add endurability. The total weight of the backpack is 35 g, which is below the recommended maximum of 2-5% of the body mass of *C. hoffmanni* (Coughlin and van Heezik, 2014; Hayssen, 2011; Peniche et al., 2011). The design of the backpack was handmade, cutting the semi-elastic false leather material considering the size and shape of the animal's body to cause the least discomfort possible. From the central square containing the tracking device in the dorsal area, the four prolongations with a 2 cm Velcro end were attached to the cardboard structure positioned in the chest area of the animal. These materials were selected due to their malleability and non-enduring weak fixation, added to the fact that the cardboard section is a biodegradable material that facilitates the release of the backpack due to degradation under moisture.

### Experimental methodology

First, the biodegradable backpack with a Tile was designed and composed. Consecutively, an innovative SBMM was applied to track two-toed sloths. This method consists of a combination of parallel transects carrying a smartphone connected to a tracking device via Bluetooth, which is attached to a backpack that wears the subject of study, in this case, a two-toed sloth. As mentioned before, apart from the costs of materials, the need for experts to use appropriately GPS satellite-based or radiotelemetry technologies can be an obstacle to implementing wildlife tracking activities. Thus, as a possible alternative, SBMM use was assessed in two scenarios: first, evaluating its detection success for non-expert biology students during a two-toed sloth monitoring; and second, quantifying the success rate of different *in situ* implementations.

To facilitate the subject's adaptation to carrying the backpack, it was cautiously introduced before their release during rehabilitation (in 3/4 occasions). In the case of Argal, the two-toed sloth was directly transferred to a nearby forest and followed-up as the animal was struggling in an urban area (1/4); therefore, no rehabilitation or acquaintance with the backpack proceeded. In the process of fitting the backpack to the body of the two-toed sloths, no drugs were administered or forceful immobilizations were performed. The researcher (R.V-B.) managed and participated in the rehabilitation of the two-toed sloths present in this study along with veterinarians from Mansion Mascota and Parque Histórico Zoo. Researchers' expertise in animal behavior, caregiver relationships, and animal recognition of the practitioner help in the moment of attaching the backpack's arms gently and carefully to the cardboard Velcro section in the chest area of the animals. Negative experiences with the handlers or, generally, humans, will produce a strong rejection to any manipulation. The process could take 10 min or 1 h depending on the animal's temperament, and attempts were abandoned when major opposition was faced to avoid stress on the animal. During the handling of the animal, signals of discomfort and distress must be recognized for protection of the animal (Morton and Griffiths, 1985). Continuous aggressive displays (hiss and slash), increase in body temperature, popped eyes or rhinarium humidity should be considered when identifying stress on the animal (Hayssen, 2011; Louw et al., 1972).

#### SBMM evaluation experiment

**Fig. 2.**
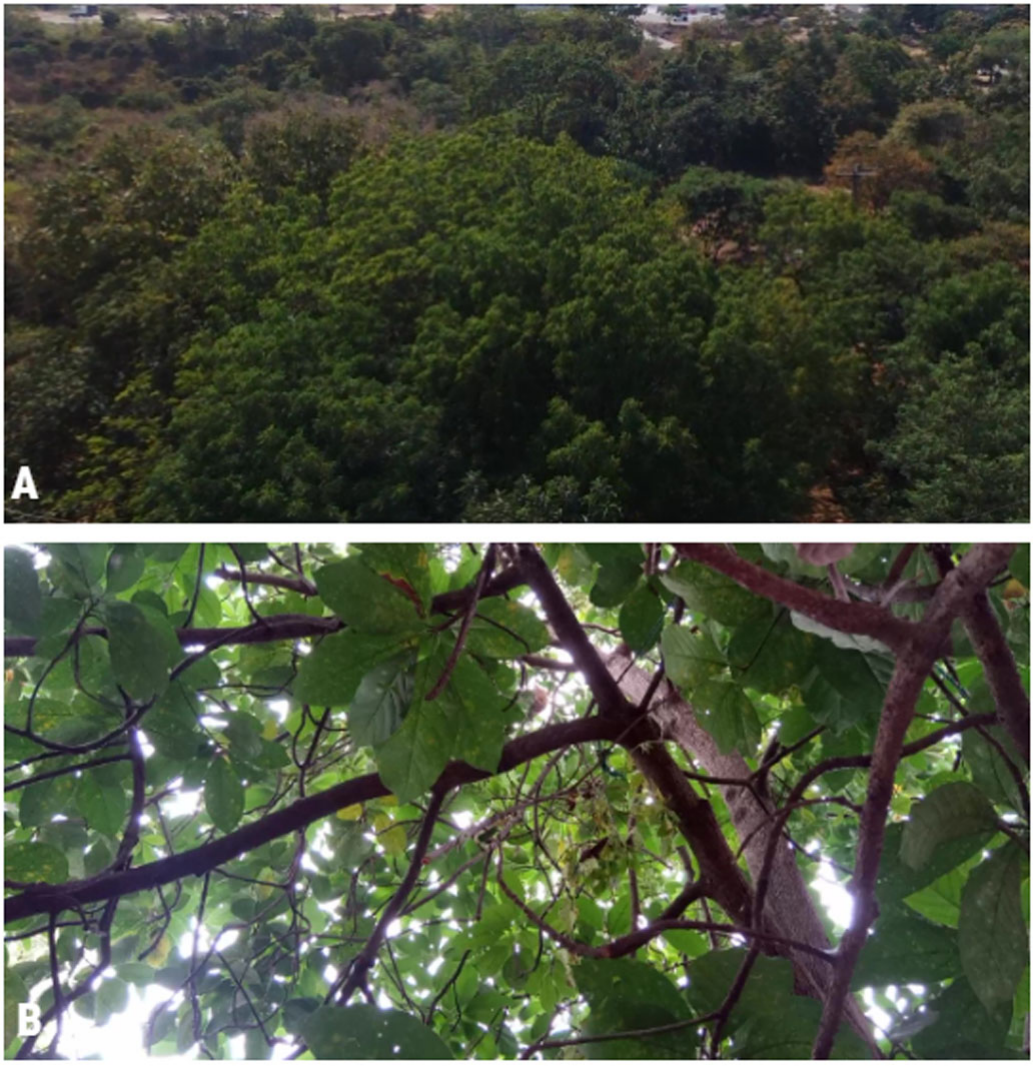
**Visual documentation of the *in situ* experimentation.** (A) Aerial image of the secondary forests in which the evaluation was conducted. (B) View of the two-toed sloth *Choloepus hoffmanni* during the search trial.

In the moment of the experiment, the two-toed sloth with the backpack was resting at 12:00 in a forested area of 647 m^2^ located in the field of ESPOL University during the dry season (Fig. 2A,B). The area is surrounded by secondary forest patches and near the Bosque Protector Prosperina (BPP). The scenario consists of 41 trees between 8 and 21 m high of different species (Fig. 2A): neem (*Azadirachta indica*), mango (*Mangifera indica*), avocado (*Persea americana*), guayabo (*Psidium guajava*), compoño (*Albizia multiflora*), cabo de hacha (*Machaerium millei*), mimosa (*Acacia dealbata*) and guasmo (*Guazuma ulmifolia*).

Eighteen biology students between 20 and 24 years of age with no experience monitoring animals participated in the experiment under informed consent. First, the presence of a two-toed sloth in the area was communicated to students, without specifying the exact location. In groups of four, they initiated the search through direct observation. They were told to perform linear transects in parallel at 5 m distance from one to each other, moving together and checking each tree to visually detect the presence of the two-toed sloth (Choperena-Palencia and Mancera-Rodríguez, 2018). In total, four groups consisting of four students followed the stated linear transects twice during the 15 min of the session by applying a visual search. Once the session was finished, without having observed their classmates and with a time limit of 15 mins, another student used the SBMM individually to locate the two-toed sloth. Then, from the four initial participants, three randomly selected members were grouped to initiate the search together again, this time using the SBMM. Once they finished, the last member of the four returned to conduct the exercise individually with the SBMM. Thus, in addition to four team visual transects, the SBMM was applied on ten occasions, including the only visual transect search for a total of 14 occasions. In total, four teams of four individuals without previous experience in the exercise tried to visually detect the animal without the SBMM, and, in contrast, four teams without previous experience, three individual searchers as a second trial (here referred to as ‘with experience’ because they tried once in the group activity), and three individual searchers without experience, applied the SBMM (Table 2). Detection results were noted through a questionnaire. It should be noted that the students were not familiar with the Tile technology or the respective app before the exercise, and experienced searchers were considered as individuals repeating the exercise in a different modality (SBMM).

Before applying the SBMM, the use of the Tile, the functioning of the Bluetooth signal, and the display on the screen reading the intensity of the signal were explained. Additionally, an explanatory note was given so that they could consult it when needed. During this activity, the students' questions were answered only by repeating the complete information of the note.

At the end of the activity, they filled out a questionnaire with the results, mentioning (1) whether they perceived the presence of the two-toed sloth, (2) their guess of the zone in which the two-toed sloth was located (in case they did not observe the animal), (3) their guess on trees in which the animal could be located (two to three trees), (4) their guess on a specific tree, and (5) if they had visually detected the two-toed sloth.

#### *In situ* implementation

The use of the SBMM was implemented individually during the follow-up and monitoring of three rehabilitated and released two-toed sloths. Two of them were rehabilitated in the veterinary specialized in wildlife Mansión Mascota (Bravo and Polita) and one in Parque Histórico (Lina). A free-roaming male two-toed sloth was monitored after a translocation (Argal) (Table 1). Bravo (Villalba-Briones et al., 2022), Polita and Argal were released in the BPP (−2° 09′ 11.09″ N, 79° 57′ 40.95″ W), in Guayaquil, and the last one (Lina) was released under supportive feeding regime in Parque Hístorico Zoo's forest (San Borondón municipality) (−2° 08′ 39.26″N, 29° 52′ 07.58″ W).

**Fig. 3.**
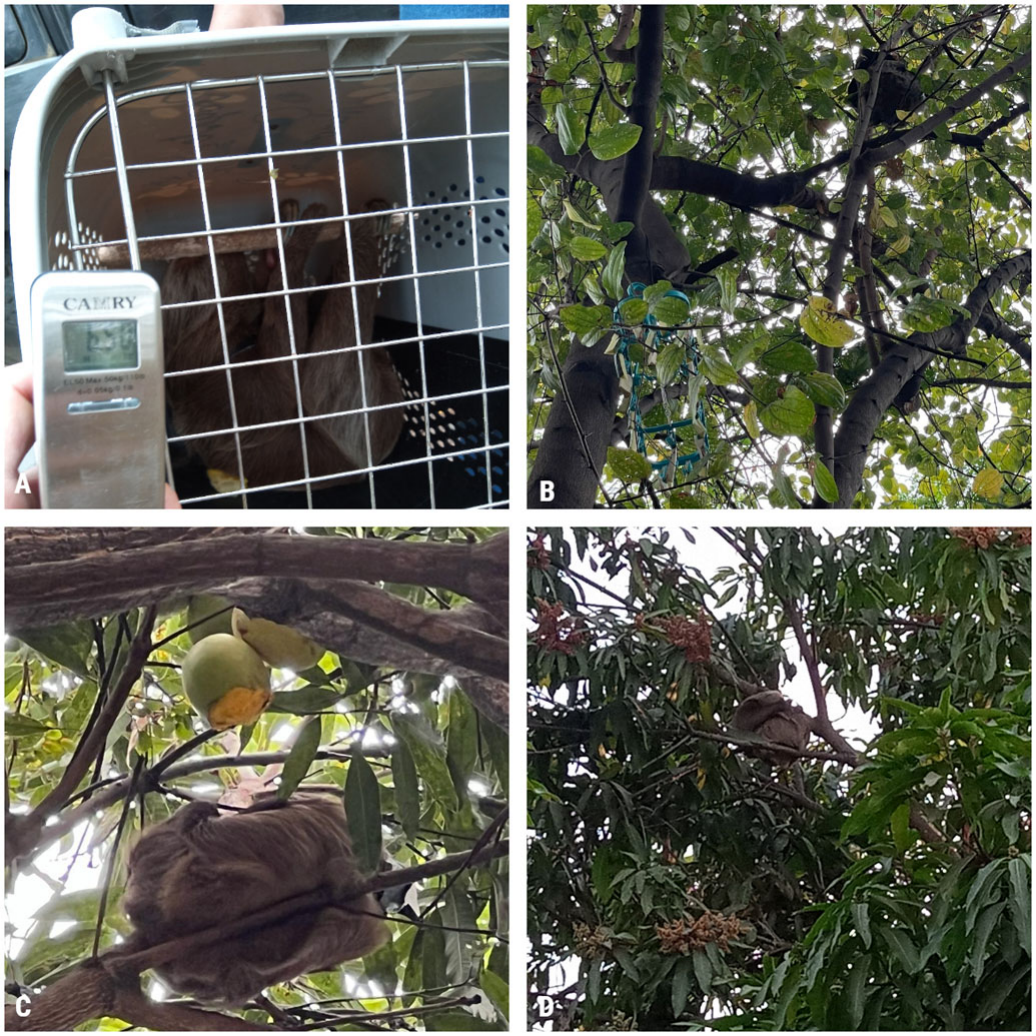
**Images of released animals during the project.** (A) Photograph of Polita with the backpack in a kennel cage adapted to allow grip and diminish the animal's vulnerability against movement during transportation. (B) Photograph of Lina showing the location of the platform for feeding support. (C) Record of independent feeding of Lina (day 35). (D) Image of the translocated wild two-toed sloth, Argal, showing the backpack lacking one of the superior attachments (day 2).

A biodegradable backpack with a Bluetooth tracker was fitted to the two-toed sloths before release during their time of rehabilitation (Fig. 3A), and responses were observed. The backpack was maintained after the release of the animal for monitoring purposes. Every monitoring day was performed by one researcher. When, due to logistic problems, the SBMM could not be implemented, a group of three observers followed the individual in the case of Lina. During follow-up activities, one of the two-toed sloths (Lina) was supported actively with zucchini, kiwi, malanga and mango fruits (Fig. 3B). During the monitoring periods, events related to the establishment process and follow-up were noted (Fig. 3B-D).
